# Development of Niosome-Entrapped Purple Waxy Corn Cobs (*Zea mays* L.) Extracts to Enhance UVB-Protection and Anti-Melanogenesis Activities

**DOI:** 10.3390/ijms262110586

**Published:** 2025-10-30

**Authors:** Inpakob Thongphachanh, Nattawadee Kanpipit, Suthasinee Thapphasaraphong

**Affiliations:** 1 Pharmaceutical Chemistry and Natural Products Program, Faculty of Pharmaceutical Sciences, Khon Kaen University, Khon Kaen 40002, Thailand; Inpakob.t@kkumail.com; 2 Department of Pharmaceutical Chemistry, Faculty of Pharmaceutical Sciences, Khon Kaen University, Khon Kaen 40002, Thailand; natawadee.k@kkumail.com

**Keywords:** purple waxy corncobs (PWCC), anthocyanin, niosome encapsulation, UV protection

## Abstract

Purple waxy corn cobs (PWCCs) represent an underutilized agricultural waste rich in anthocyanins with promising cosmeceutical potential. This study investigated niosome-based encapsulation to enhance the stability and bioactivity of PWCC anthocyanin extracts. PWCC extract was macerated in 50% ethanol. The extract exhibited a high total anthocyanin content (3.02 ± 0.81 mg C3GE/L), while cyanidin-3-glucoside identified as the major anthocyanin (1.17 ± 0.02 mg/g dry weight). Furthermore, the extracts showed strong antioxidant activities as evidence by DPPH, ABTS, and FRAP assays. The optimized niosome preparations synthesized by the probe sonication method exhibited better entrapment efficiency (80–85%), nanoscale particle size (185–296 nm), and stable zeta potential (−29 to −32 mV). TEM verification of the spherical morphology and FT-IR spectra confirmed the successful loading of anthocyanins. The thermal stability test exhibited negligible changes in the particle size and zeta potential. Furthermore, in vitro release profile followed the Higuchi model, indicating enhanced release kinetics. Biological assays demonstrated moderate UVB protection effects and potent anti-melanogenesis activity in B16F10 cells. Notably, formulation N5 exhibited the highest tyrosinase inhibition and melanin synthesis suppression. These findings indicate that niosome-based encapsulation represents a promising strategy for enhancing the stability, bioavailability, and biological efficacy of anthocyanin extracts, especially in the cosmetic and pharmaceutical industries.

## 1. Introduction

Purple Waxy Corn (*Zea mays* L.) contains a high content of anthocyanins, bioactive pigments with inherent health value and varied uses in food, cosmetics, and pharmaceuticals. Among them, one that has been bred at the Plant Breeding Center, Faculty of Agriculture, Khon Kaen University, has a superior anthocyanin content, especially in corn cobs [1]. This compound has been thoroughly documented as having the ability to boost not only the nutritional value and biological characteristics of corn but also as an opportunity for extraction and utilization in health-oriented products [2]. One of the outstanding abilities of corn, such as those contributing to UV sensitivity, can be utilized positively owing to the presence of anthocyanins and inherent UV protectants. Interestingly, anthocyanins from Purple Waxy Corn are natural UV protectants that can limit oxidative damage to plant tissue. This ability can enable the development of UV-protective ingredients for cosmetic and skin-care applications, as well as stabilize natural light-resistant food colorings. In addition, anthocyanins can potentially be used as natural and environmentally friendly alternatives in the food and packaging sectors. Anthocyanins are flavonoids of the subclass and display a range of biological activities, such as antioxidant activity [3], anti-inflammatory [4], anticancer [5], and UVB-protective properties [6]. In purple waxy corn, Cyanidin-3-glucoside, Pelargonidin-3-glucoside, and Peonidin-3-glucoside anthocyanins are present in their derivatives, malonyl and ethylmalonyl-glucosides [7]. Anthocyanin sources differ in their composition and usability. Berries yield multicomponent anthocyanin profiles with intense bioactivities but are restricted by being expensive, seasonal, and having limited shelf life [8]. Red cabbage yields stable acylated anthocyanins and is used extensively as a food colorant, but it has an offensive odor and pH-dependent color, limiting its widespread use [9]. Purple waxy corn cobs (PWCCs) typically yield high levels of cyanidin-based anthocyanins with high antioxidant and UV protection capacities. As a crop by-product, PWCC enables economic and environmentally friendly recovery and offers an accessible alternative to imported sources, which is far more desirable for food, cosmetic, and pharmaceutical purposes [6]. Although different anthocyanins have specific medicinal relevance, they are highly sensitive to environmental factors such as heat, light, metal ions, and pH conditions, and are unstable during storage and processing. One of the suggested means of stabilizing anthocyanins and enabling controlled release is the encapsulation of anthocyanins in nanoscale carriers [10]. Moreover, with such potential uses, further studies on the stabilization and delivery of anthocyanins, specifically through nano-encapsulation processes, are needed. This is to unlock their full functional value in both industry and health [11].

Niosomes contain only nonionic surfactants, which are either polymeric or nonpolymeric. Niosomes possess desirable characteristics such as biocompatibility, biodegradability, non-toxicity, non-immunogenicity, non-carcinogenicity, and stability against hydrolysis, which are largely influenced by their bilayer composition and method of preparation [12]. The preparation of niosomes is the choice of suitable methods based on required characteristics such as vesicle size, size range, bilayer composition, entrapment in the water phase, and permeability through the membrane, most commonly using an aqueous vehicle with the optional miniaturization of size steps [13]. Sonication is an effective way to obtain small homogeneous vesicles [14]. Niosomes effectively release drugs over prolonged time intervals and enhance drug penetration at the target sites [15]. Niosomes are used for photoprotection, sunscreen stabilization, and controlled release [12]. Niosomes would also improve sunscreen retention within the skin, reducing the chances of toxic uptake by the bloodstream. By remaining on the skin surface, niosomes can retain more ultraviolet radiation in the bay and potentially reduce the risk of skin cancer [12].

Anthocyanin extracts from other natural sources have been shown to exhibit photoprotection and anti-melanogenic activity. For instance, an extract of *Vaccinium uliginosum* was observed to inhibit UV-induced skin injury in hairless mice by stimulating skin hydration, inhibiting transepidermal water loss, inhibiting MMP gene expression, and stimulating TIMPs and antioxidant-related gene expression. In addition, it also suppresses UVB-induced activation of the MAPK signal transduction pathway, that is, ERK, JNK, and p38, and their corresponding inflammatory cytokines [16]. Likewise, anthocyanins isolated from *Hibiscus syriacus* L. suppressed melanin production by suppressing α-MSH-induced activity and downregulating important melanogenesis-related genes such as tyrosinase and MITF. Fermented black rice also suppressed melanin production in B16F10 cells by inhibiting tyrosinase and related proteins [17]. Evidence for this result is that anthocyanin-dense rose extract can inhibit tyrosinase and melanin production without cytotoxicity. In addition, clinical trials have confirmed that the application of rose extract lightens the skin by enhancing skin luminosity [18], these findings demonstrate the cosmetic importance of anthocyanins as the active ingredients. Although the cosmetic potential of natural sources of anthocyanins has been extensively explored, that of purple waxy corn cob (*Zea mays* L.) is relatively underexplored. Therefore, we would like to add value to create maximum benefits from cob by-products. Our study aimed to formulate a niosome delivery system incorporating purple waxy corn cob extracts. This step was also designed to increase the stability and effectiveness of the extract, especially its UVB protection and anti-melanogenesis activity, thus expanding its range of applications in cosmeceutical products.

## 2. Result

### 2.1. Extraction Results of Anthocyanin and the Composition from Purple Waxy Corn Cobs

The yield of PWCC extract (according to the process in Figure S1) was 6.34%. The total anthocyanin content was 3.02 ± 0.81 mg C3GE/L. Antioxidant assays showed IC_50_ values of 72.32 ± 9.71 µg/mL for DPPH, 17.78 ± 0.57 µg/mL for ABTS, and a FRAP value of 4.21 ± 0.10 mg Fe (II)/g are summarized in Table 1. Moreover, individual anthocyanin was analyzed by HPLC-MS/MS analysis and the operating conditions were validated according to a previous report [19]. This analytical method was adapted from a previously validated procedure reported to ensure reliability and reproducibility of the measurement [19]. The validation parameters are summarized in Table S1, and the anthocyanin chromatogram is shown in Figure S2 and compared with the standard curve of cyanidin-3-glucoside shown in Figure S3. In this research, the major compound, cyanidin-3-glucoside, was obtained at 1.17 ± 0.02 mg/g dry weight [20].

### 2.2. The Characterization of Niosome Formulation

#### 2.2.1. Entrapment Efficiency, Particle Size, Polydispersity (PDI), and Zeta Potential

A total of five niosomal formulations were developed, as presented in Figure S4. The entrapment efficiency, particle size, polydispersity index (PDI), and zeta potential of niosome formulations prepared using the sonication method are summarized in Table 2. Among the five formulations of niosomes, they demonstrated an entrapment efficiency exceeding 80%, with values falling within the range of 80–85%. The chosen formulations were further studied for particle sizes between 185.60 nm and 296.50 nm. PDI was less than 0.5, which was between 0.14 and 0.42, showing a narrow size distribution. The zeta potential values were also less than −30 mV, between −29.30 and −32.90 mV.

#### 2.2.2. Transmission Electron Microscopy (TEM) Measurement

**Figure 1:**
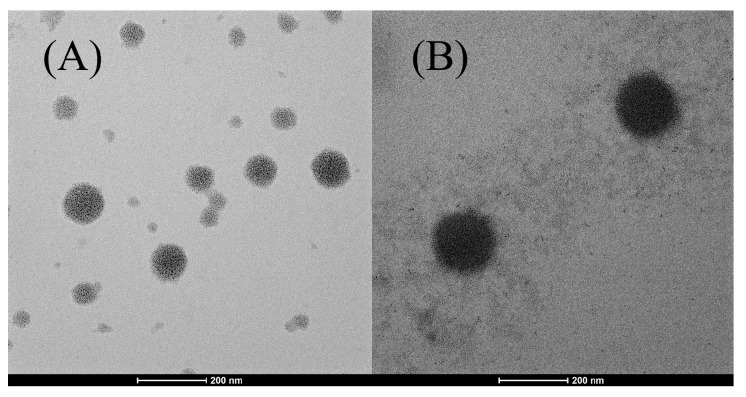
Morphology of niosome formulations prepared using the sonication method, observed through transmission electron microscopy (TEM): (**A**) niosome blank formulation (N3) at a scale bar of 200 nm, (**B**) niosome-encapsulated PWCC extract (N5) at a scale bar of 200 nm.

The morphology of the niosomes was confirmed by morphological analysis. Among the formulations, the extract-loaded niosome formulation (N5) showed the highest entrapment efficiency and was selected for TEM determination compared to blank niosomes (N3). Figure 1 displayed the morphology of N3 and N5, showing a spherical with the average size around 100–200 nm. The blank niosomes exhibited the smaller particle size with pale and translucent, indicating the absence of f encapsulated material. The PWCC extract-loaded niosomes exhibited a lager particle size and darker compared to empty niosomes, confirming the successful encapsulation of the extract. The observed morphological uniformity aligns with earlier studies on spherical vesicle formation in anthocyanin-loaded niosomes [21].

#### 2.2.3. Stability Evaluation

**Figure 2:**
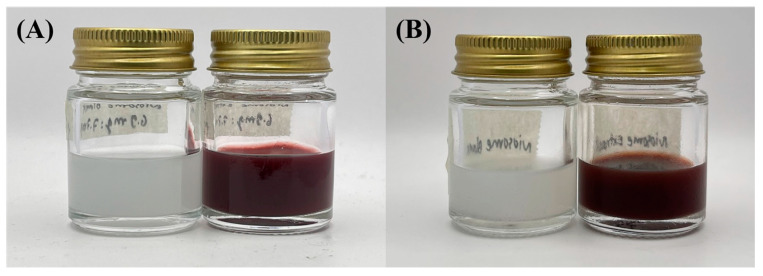
Stability of the niosome formulation during heating–cooling storage. (**A**) Niosome blank (N3) and niosome entrap 1%PWCC extract (N5) before storage, and (**B**) niosome blank and niosome entrap 1%PWCC extract after storage.

The findings of this study indicate that the stability profile of the niosome formulations was satisfactory under heating–cooling storage. The selected formulation for the active extract-loaded niosome was N5, with a formulation of (1% PWCC extract), and the blank niosome was N3. There is a visible change in color, particularly in formulation N5, which is likely due to the effect of heat, oxidative stress, and possibly pH changes during storage, resulting in the degradation of anthocyanins and a reduction in color intensity [22], as illustrated in Figure 2. Entrapment efficiency was not statistically different before and after storage (*p* < 0.05), suggesting that the active ingredients were in good entrapment within the vesicles. However, notable and statistically significant changes in the particle size, polydispersity index (PDI), and zeta potential (*p* < 0.05) before and after storage suggest some degree of physical instability (Table 3). The observed increase in size and PDI serves as direct evidence of niosome aggregation or fusion, wherein individual vesicles cluster or merge to form larger, less homogeneous particles. Although a higher zeta potential usually indicates greater colloidal stability, an increase in this value along with aggregation also suggests a reorganization of the surface charge when vesicles interact and merge [23].

#### 2.2.4. FT-IR Spectroscopy

The FT-IR spectrum of purple waxy corn cob extract (PWCC) (Figure S5A), which exhibited functional groups and wavenumbers between 400–4000 cm^−1^ which were identified as OH stretching (3289.84 cm^−1^), C-H functional group stretching (2920.86 cm^−1^), C=O stretching (1702.86 cm^−1^), C=C stretching (1598.79 and 1515.83 cm^−1^), C-C stretching (1451.82 and 1417.80 cm^−1^), CH_2_ bending (1370.80 and 1333.80 cm^−1^), C-O (1259.75, 1163.74 and 1028.71 cm^−1^) and C-H bending (817.27, 774.17 and 514.36 cm^−1^) [24,25].

The FT-IR spectrum of blank niosome formulation (N3) shows the typical vibrations of the functional groups (Figure S5B). An extensive absorption at 3316.62 cm^−1^ for O–H stretching, along with C–H stretching peaks at 2971.79, 2930.78, 2877.81, 2376.96, and 2319.96 cm^−1^ were observed. The presence of C=O stretching vibration was confirmed by the peak at 1657.90 cm^−1^. The C–C stretching was observed at 1460.79 and 1414.77 cm^−1^, while CH_2_ bending vibrations were observed at 1377.74 and 1330.78 cm^−1^. Several absorption bands related to C–O stretching were detected were observed at 1284.78, 1229.80, 1135.65, 1076.61, and 1039.34 cm^−1^. C–H bending vibrations were also evident at 989.46, 920.96, 836.85, 802.67, 520.74, and 473.34 cm^−1^, which explained the complicated molecular interactions in the blank niosome.

The FTIR spectrum of the PWCC extract encapsulated niosomes (N5) showed vibrational bands corresponding to various functional groups (Figure S5C). A broad band at 3317.62 cm^−1^ signified O–H stretching, whereas peaks at 2971.78, 2930.78, 2875.70, and 2349.96 cm^−1^ indicated C–H stretching vibrations.

The existence of C=O stretching was indicated through the emergence of a band at 1648.90 cm^−1^. The C–C stretching vibrations were observed at 1460.77 and 1417.76 cm^−1^, while CH_2_ bending was observed at 1376.72 and 1331.75 cm^−1^. The C–O stretching bands were identified at 1284.76, 1227.78, 1135.60, 1076.57, and 1039.30 cm^−1^. The C–H bending vibrations were also evident at 989.45, 921.15, 836.75, 801.27, 520.54, and 474.74 cm^−1^.

The FT-IR spectrum of Span 20 exhibited the characteristic absorption bands of different functional groups (Figure S6A). A broad peak at 3392.86 cm^−1^ was assigned to O–H stretching, whereas peaks at 2922.60, 2853.69, and 2376.96 cm^−1^ were assigned to C–H stretching vibrations. High absorption at 1738.66 cm^−1^ was assigned to C=O stretching, whereas a peak at 1560.93 cm^−1^ was assigned to C=C stretching. A signal at 1462.79 and 1418.83 cm^−1^ was allotted to C–C stretching, while CH_2_ bending vibrations were found at 1376.80 and 1303.80 cm^−1^. The bands for C–O stretching were found at 1235.74, 1165.65, and 1060.57 cm^−1^. Some of the peaks between 978.70 and 462.36 cm^-1^ were also allotted to different C–H bending vibrations, which indicates that there are aliphatic chains, and which justifies the structure elements of Span 20 [26].

The FTIR spectrum of cholesterol displayed the absorption bands according to its molecular structure (Figure S6B). The high-intensity peaks at 3427.90 cm^−1^ attributed to O–H stretching, along with four bands at 2930.71, 2900.76, 2867.76, and 2847.78 cm^−1^ corresponding to C–H stretching vibrations. The absorption band at 1671.94 cm^−1^ corresponded to C=O stretching. Peaks at 1463.80 and 1436.83 cm^−1^ were attributed to C–C stretching, while CH_2_ bending vibrations were noted at 1377.82, 1365.82, and 1333.87 cm^−1^. The C–O stretching region exhibited numerous bands at 1273.91, 1236.89, 1190.89, 1169.90, 1131.89, 1083.90, 1054.71, 1022.81, and 1008.89 cm^−1^. Moreover, a series of bands due to C–H bending was identified at 954.84, 926.68, 881.90, 839.76, 799.58, 739.98, 689.68, 625.88, 591.98, 571.78, 501.38, and 465.28 cm^−1^, supporting the occurrence of aliphatic and steroidal moieties within the cholesterol structure [26].

**Figure 3:**
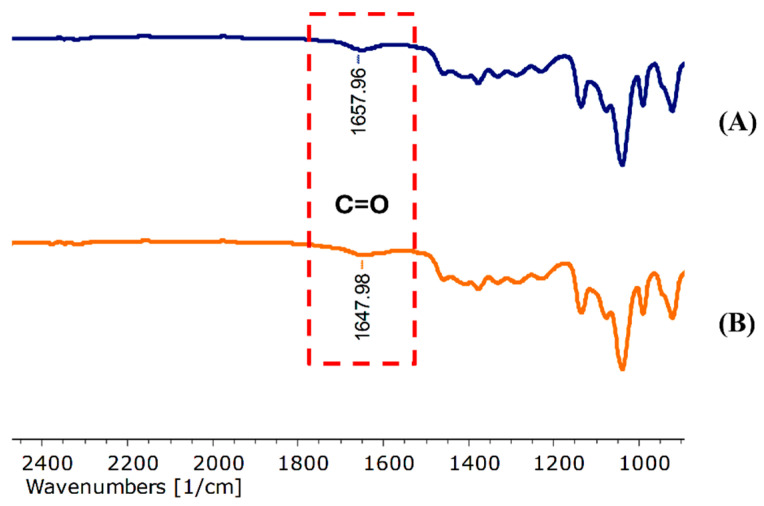
Spectral interactions between carbonyl functional groups. (**A**) Niosome blank, N3 and (**B**) niosome encapsulating PWCC extract, N5.

The FT-IR spectra of the blank niosomes, N3 (Figure S5B) and PWCC extract-loaded niosomes, N5 (Figure S5A) indicated the presence of characteristic functional groups. The presence of Span 20 was provided through C–C stretching bands at 1460.79 cm^−1^ in blank niosome and 1460.77 cm^−1^ in PWCC niosomes. The C–H stretching at 2930.78 cm^−1^ and C–O stretching bands at 1135.65 cm^−1^ and 1135.60 cm^−1^ were typical of cholesterol [27]. The FT-IR spectra revealed a notable shift in the C=O stretching vibration from 1657.96 cm^−1^ in the blank niosome (N3) to 1647.98 cm^−1^ in niosome encapsulating PWCC extract (N5) in Figure 3. Furthermore, Figure S7 presents the FT-IR spectra of Span 20, cholesterol, and propylene glycol for comparative analysis. This alteration indicates potential interactions between the carbonyl groups of the extract and the components of niosome membrane, indicating the successful incorporation of the active compound into the vesicular structure [28]. 

#### 2.2.5. In Vitro Release Study

**Figure 4:**
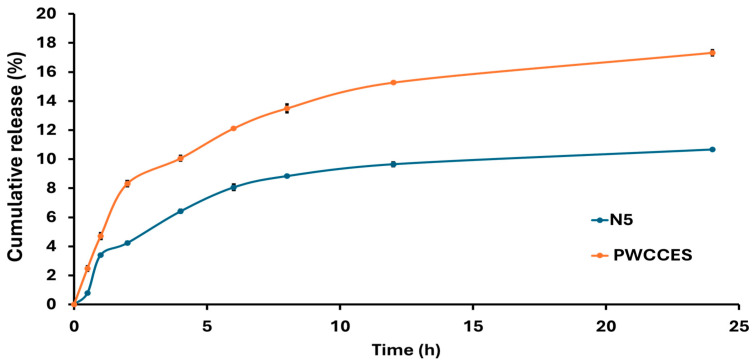
Comparison of Niosome formulation release profiles from niosome-encapsulated PWCC extract (N5) and PWCC extract solution (PWCCES).

The release profile shown in Figure 4 indicates that the niosome-encapsulated PWCC extract formula (N5) achieved a cumulative release of 10.66% after 24 h, whereas PWCC extract solution (PWCCES) achieved a higher value of 17.32% at the same time. Although the extract solution showed higher and faster release, the niosome formulation showed a more controlled and long-lasting release profile. Kinetic analysis presented in Table 4 revealed that N5 (Figure S8) and PWCCES (Figure S9) presented the highest correlation coefficients (R^2^ = 0.9901 and 0.9662, respectively) for Higuchi model, indicating that both followed a diffusion-controlled release mechanism. The niosome formulation (N5) demonstrated a gradual diffusion of anthocyanins through the hydrated niosomal bilayers.

In addition, release rate constant (K_p_) obtained from Korsmeyer–Peppas model, represents the overall rate at which the active compound diffuses or is released from the niosomal matrix. Therefore, K_p_ of N5 and PWCCES were calculated as 3.2636 and 5.1713, respectively (Table 4). A higher K_p_ value of PWCCES indicates a faster release rate of the entrapped anthocyanins, whereas N5 showed a lower K_p_, suggesting a slower and more sustained release behavior. Therefore, K_p_ serves as indicator of release performance among different formulations, reflecting how formulation parameters influence drug diffusion through the vesicular system. Furthermore, the release exponent(n) obtained from Korsmeyer–Peppas model describes the mechanism of drug release from delivery system. In this study, n value of N5 (0.4838) was close to 0.5, indicating the release of anthocyanins followed Fickian diffusion mechanism.

This finding is consistent with previous reports describing diffusion-dominated release behavior from non-ionic surfactant vesicles. However, the findings illustrate that while free extract yields instant release, the niosome system yields sustained release, improving stability, bioavailability, and therapeutic activity of the encapsulated anthocyanins [29].

#### 2.2.6. The Biological Activities of Niosome Formulation

##### UVB Protection

The cytotoxicity of the niosome preparations and PWCC extract solution against HaCaT cells was compared using the MTT assay. HaCaT cells were treated with the PWCC extract solution and niosome formulations for 24 h. No cytotoxic cells were observed in any of the samples investigated under these conditions, as evidenced by a cell viability of >80%.

**Figure 5:**
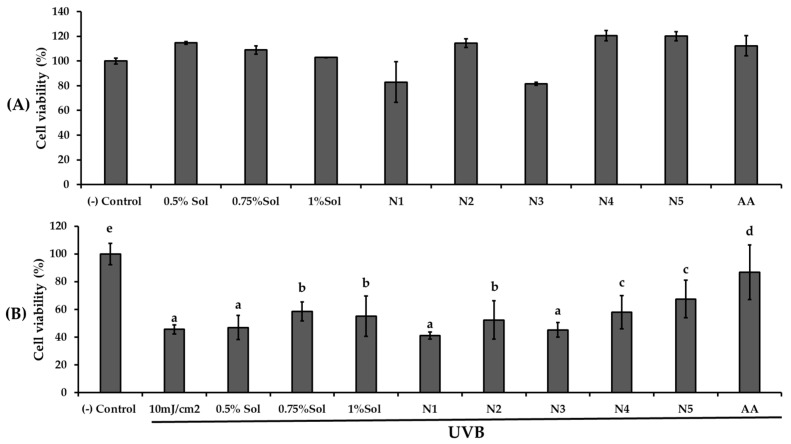
The result of (**A**) cytotoxicity of HaCaT cells with different concentrations of PWCC extract solution (0.5, 0.75, 1.0%), niosome formulations (N1–N5), and 100 µg/mL ascorbic acid (AA), and (**B**) Viability of HaCaT cells exposed to UVB radiation (10 mJ/cm^2^) with different concentrations of PWCC extract solution (0.5, 0.75, 1%), niosome formulations (N1–N5), and 100 µg/mL ascorbic acid (AA). Statistical significance was determined using Duncan’s test (*p* < 0.05), where different letters (a–d) denote significant differences among the groups for UVB protection. The (−) control was non-treated cells.

UVB protective effects were examined by exposing HaCaT cells to 10 mJ/cm^2^ of UVB radiation after 24 h pretreatment with PWCC extract solution, ascorbic acid, and their niosome formulations (N1, N2, N3, N4, N5), as shown in Figure 5.

Pre-treatment with PWCC extract solution and niosome formulations N2 (0.5% PWCC extract), N4 (0.75% PWCC extract), and N5 (1.0% PWCC extract) notably enhanced UVB protection in HaCaT cells (*p* < 0.05) when compared to the untreated UVB-exposed control group. Niosome formulations containing PWCC extract at concentrations of 0.5%, 0.75%, and 1.0% (N2, N4, and N5, respectively) showed superior protective effects against UVB-induced cytotoxicity than the corresponding 0.5%, 0.75%, and 1.0% PWCC extracts (*p* < 0.05). Among all formulations, N5 containing 1.0% PWCC extract exhibited the highest level of UVB protection. However, the UVB protective effects of all niosomal formulations were still lower than the positive control (ascorbic acid) and the negative control.

##### Anti-Melanogenesis

The cytotoxicity of B16F10 cells pretreated for 48 h with varying concentrations of the PWCC extract solution and its niosome formulations were evaluated using the MTT assay. All formulations were further screened for anti-melanogenesis potential. B16F10 cells were incubated with PWCC extract solution and niosome formulations for 48 h. The cells were treated and stimulated with α-melanocyte-stimulating hormone (α-MSH).

**Figure 6:**
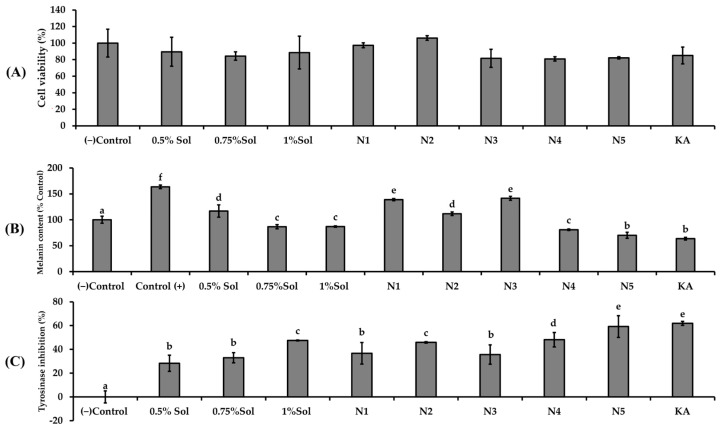
The effects of (**A**) different concentrations of PWCC extract solution, niosome formulations (N1–N5), and kojic acid (KA)100 µg/mL on B16F10 melanoma cells stimulated by α-melanocyte-stimulating hormone (α-MSH) were assessed in terms of melanin content (**B**) and tyrosinase inhibition (**C**). The control group consisted of untreated α-MSH-stimulated B16F10 melanoma cells. Data are presented as the mean ± SD from three independent replicates. Statistical significance was determined using Duncan’s test (*p* < 0.05), where distinct letters (a–f) denote significant differences among groups for melanin content and letters (a–e) indicate significant differences among groups for tyrosinase inhibition. (−) Control is non treated cells.

The results in Figure 6A indicated that none of the niosome formulations (N1–N5) exhibited cytotoxic effects on B16F10 cells. The results of melanin content reduction (Figure 6B) and tyrosinase inhibition (Figure 6C) appear to exhibit a dose dependent relationship with the concentration of PWCC extract. The niosome formulations containing PWCC extract (N2, N4, N5) demonstrated reduced melanin content and increased tyrosinase inhibition compared to blank niosome (N1 and N3).

Additionally, all niosome encapsulating PWCC extract exhibited enhanced the anti-melanogenesis effects compared to the same concentration of PWCC extract alone. Among all samples, N5 exhibited the lowest melanin production and the most significant inhibition of tyrosinase activity (Figure 6C), indicating significantly strong potential for anti-melanogenesis as well as kojic acid (positive control). The observed difference was attributed to the properties of the N5 formulation which contained the highest concentration of PWCC extract (1%PWCC extract). This formulation achieves the greatest entrapment efficiency of 85.74 ± 1.19%, in comparison to N2 (0.5% PWCC extract) and N4 (0.75%PWCC extract), which exhibited entrapment efficiency at 84.88 ± 2.11 and 83.68 ± 3.18%, respectively (Table 2).

## 3. Discussion

### 3.1. The Extraction of Anthocyanin and the Composition from Purple Waxy Corn Cobs

From the extraction result in Table 1, PWCC extracted by 50% ethanol exhibited total anthocyanin at 3.02 ± 0.81 mg C3G/g dry weight, while a previous research studied on PWCC extracted by 50% ethanol exhibit a higher content of anthocyanin at 7.46 ± 0.05 mg C3G/100 g [1]. Furthermore, recent research exhibited cyanidin-3-glucoside in purple waxy corn cobs extracted with 50% ethanol at 1.17 ± 0.02 mg/g dry weight, whereas the previous research reported that was the predominant anthocyanin identified in purple waxy corn cobs extracted with 50% ethanol, with quantified content of 2.42 ± 0.03 mg/g dry weight. In addition, the current results exhibited relatively lower antioxidant activity compared to the previous report, particularly in the FRAP assay, where the current research showed a FRAP value of 4.21 ± 0.10 mg Fe(II)/g, in contrast to 595.11 ± 1.31 mg Fe(II)/g reported previously [1]. This difference can be explained by variations in raw material purity and storage conditions, because anthocyanins are prone to degradation during storage and drying. Powder PWCC and dried PWCC were used in our research to make it reproducible and for long storage, which could have been at the expense of the stability of anthocyanin. In addition, variability in extraction conditions (e.g., time, temperature, and solvent-to-solid ratio) and natural variation in crop yields may also be responsible for the low yield. Despite these limitations, 50% ethanol was identified as the most effective solvent system in this study, yielding the highest extraction efficiency and anthocyanin recovery while ensuring safety for applications in food and cosmetic applications [1].

### 3.2. The Characterization of Niosome Formulation

#### Entrapment Efficiency, Particle Size, Polydispersity (PDI), and Zeta Potential

Among the 5 prepared niosome formulations, three exhibited encapsulation efficiencies exceeding 80%. Based on this criterion, these three formulations were selected for the subsequent analysis of particle size characteristics. This represents a high entrapment efficiency (EE) and demonstrates the effectiveness of vesicles for the entrapment of anthocyanins. High EE is very important to demonstrate adequate drug loading in proving that there is sufficient loading of the drug and minimization of the loss of active constituents. More concentrated EE formulations were chosen for subsequent studies to verify their potential for further development as delivery systems [29].

The particle size of the niosome formulations ranged from 185 to 296 nm, placing them within the optimal nanometer range for topical and transdermal administration. These particles can easily interact with the stratum corneum, thus possibly enhancing drug deposit into the skin without irritation or disruption of the barrier. Additionally, small particle sizes facilitate improved cellular uptake and penetration [30].

The polydispersity index (PDI) was 0.14 to 0.42 below the critical value of 0.5 and had a monodisperse and homogeneous particle size distribution. This is an outstanding quality for vehicle-based systems as it reflects uniform formulation and predicts stable performance when stored and used [30].

Further, the zeta potential of the chosen formulations ranged between −29.30 and −32.90 mV, close to or above the threshold value of ±30 mV generally believed to be required for colloidal stability. A high absolute zeta potential inhibits vesicle aggregation due to electrostatic repulsion and leads to long-term physical stability of niosome dispersions [31].

Overall, all niosome formulations were within acceptable limits, had good physicochemical stability, consistent particle size distribution, and excellent encapsulation efficiency for anthocyanins isolated from purple waxy corn cobs. These characteristics make them suitable for further development, especially for use in dermal or cosmetic delivery systems [32].

### 3.3. Stability Evaluation

Stability studies revealed that although entrapment efficiency was not affected by thermal cycling, particle size and PDI increase showed vesicle agglomeration and loss of homogeneity. Physical instability influences long-term delivery by potentially changing the release profile, skin penetration, and overall formulation stability after storage. Aggregated vesicles are most likely to be slower or less reliable for drug delivery and can decrease bioavailability in transdermal systems. However, a uniformly high EE ensures that the vesicle core organization is maintained, which in turn safely encapsulates the inner active extract. Thus, although the system is chemically stable against anthocyanins, optimization approaches such as surface modification and addition of cryoprotectants or antioxidants may be required to avert aggregation and provide long-term physical stability for effective utilization, as summarized in (Table 3).

### 3.4. In Vitro Release Study

Release studies in Table S2 demonstrated that the cumulative release of the niosome encapsulated PWCC extract (N5) was relatively low (10.66% over 24 h) compared with the free extract solution (17.32%). However, the relatively low percentages cumulative release observed should not be interpreted as incomplete release, but rather as evidence of the niosomes (N5) provided high entrapment efficiency and their ability of controlled drug release compared to the free extract solution (PWCCES). The kinetic parameters for anthocyanin release obtained from each model are presented in Table S3. Due to its stability, the surfactant–cholesterol bilayer effectively acts as a barrier, slowing drug diffusion and producing the desired sustained release kinetics. It is important to recognize that in vitro release studies are conducted under simplified experimental conditions that do not fully reflect the complexity of biological systems. In an in vivo environment, additional factors including enzymatic degradation of the niosomal structure and active interactions with biological membranes are expected to significantly enhance both drug release and permeation across physiological barriers. Consequently, despite the modest in vitro release rates, the formulation is anticipated to substantially improve the bioavailability of the active compound through its capacity for prolonged systemic retention and targeted delivery [10].

### 3.5. The Biological Activities of Niosome Formulation

Biological studies with HaCaT cells under UVB protection demonstrated that N5 (1.0% PWCC extract) significantly showed the highest UVB protection among all formulations compared with the 1.0% PWCC extract solution alone. However, the overall UVB protection of all niosome formulations was lower than that of ascorbic acid. This suggests that the UV protection capacity changes in terms of durability rather than intrinsic strength. The enhancement of lightfastness through noisome encapsulation did not directly increase the UV absorption ability of anthocyanins. Rather, it retards photodegradation, hence maintaining its molecular stability and its ability to protect against UV radiation for a prolonged period. However, free anthocyanins not only become colorally unstable but also lose their UV protection capacity within a very short time after exposure [33]. Encapsulation of niosomes maintains the UV protective activity of purple waxy corn anthocyanins for a longer period. There were also, according to previous studies, anthocyanins that shielded HaCaT cells from UVB-induced damage by triggering Nrf2, elevating antioxidant enzymes, decreasing ROS, promoting DNA repair, and preventing apoptosis [34]. Furthermore, the N5 formulation also demonstrated the greatest inhibition of melanin production and tyrosinase activity, comparable to kojic acid. Although the overall UVB protection of all niosome formulations was lower than that of ascorbic acid, N5 exhibited strong anti-melanogenesis potential. These findings are consistent with previous reports showing that anthocyanins and their degradation products from *Solanum tuberosum* L. possess notable tyrosinase inhibitory effects, supporting their potential use in skin-whitening and pigmentation-control applications [35].

## 4. Materials and Methods

### 4.1. Materials

Span20 from Sigma-Aldrich (Burlington, MA, USA). Cholesterol from CARLO ERBA Reagents S.A.S. (Val-de-Reuil, France). Propylene glycol from Elago Enterprises Pty Ltd., Kemaus (Cherrybrook, New South Wales, Australia). Sodium acetate (CH_3_COONa), potassium chloride (KCl), disodium hydrogen phosphate (Na_2_HPO_4_), potassium dihydrogen phosphate (KH_2_PO_4_), and sodium chloride (NaCl) were purchased from Ajax FineChem Pty, Ltd. (Taren Point, New South Wales, Australia). Ethanol from RCL Labscran (Samut Prakan, Thailand). 3-[4,5-dimethylthiazol-2-yl]-2,5-diphenyltetrazolium bromide (MTT) was obtained from Thermo Scientific (Waltham, MA, USA). Tyrosinase, N-[3-(2-furyl) ac-ryloyl]-Leu-Gly-Pro-Ala (FALGPA) was purchased from Merck KGaA (Darmstadt, Germany). Fetal bovine serum (FBS), penicillin–streptomycin, 0.25% trypsin-EDTA, and Dulbecco’s Modified Eagle’s Medium (DMEM) were obtained from Gibco, Thermo Fisher Scientific (Waltham, MA, USA).

### 4.2. Preparation of Extracts

#### 4.2.1. The Cyanidin-3-Glucoside Content by HPLC Determination

Cyanidin-3-glucoside (C3G) content was quantified by HPLC–MS/MS using an API 3200 triple quadrupole mass spectrometer coupled with an HP 1100 binary HPLC system and Poroshell 120 SB-C18 column (4.6 × 75 mm, 2.7 µm, Agilent Technologies, Santa Clara, CA, USA). The mobile phases consisted of 5% formic acid (A) and methanol (B) with a gradient elution from 20% to 100% B over 15 min, followed by re-equilibration. The flow rate was 0.3 mL/min, column temperature 30 °C, and injection volume 10 µL. MS parameters were set according to a previous report [19]. The method validation using linearity, precision, accuracy, and recovery was performed. The extracted samples were subjected to HPLC-MS/MS and the C3G contents were determined compared with standard curves.

#### 4.2.2. Extraction of Anthocyanin from Purple Waxy Corn Cobs

Purple corn cobs (*Zea mays* L.) from the Plant Breeding Center, Faculty of Agriculture, Khon Kaen University, were air-dried and ground into a fine powder with a grinder. The powder was extracted with 50% ethanol at a ratio of 1:10 with the maceration method for 24 h at a shaking incubator (Kuhner SHAKER X, Birsfelden, Basel, Switzerland) at 25 °C and 120 rpm. This extraction process was performed three times consecutively to ensure an optimal yield. After the first extraction, the cob powder was re-extracted, followed by filtration through a thin white cloth, and subsequently through paper filter no. 1 to obtain a clear Purple Waxy Corn Cob extract (PWCCES). The solvent was evaporated under vacuum using a rotary evaporator (ONiLAB VC100, Republic of Korea) and the extract was freeze-dried (SCANVAC, Allerød, Hovedstaden, Denmark). The total anthocyanin content of the final extract was quantified by the pH-differential method [2], while the predominant anthocyanin (C3G) was determined by HPLC-MS/MS following a previously described method [19]. Antioxidant activities were evaluated using the 2,2-Diphenyl-1-picrylhydrazyl (DPPH) [36], 2,2′-azino-bis (3-ethylbenzothiazoline-6-sulfonic acid (ABTS) [36], and Ferric Reducing Antioxidant Power (FRAP) assays [37].

(1) $$\mathrm{Total}\;\mathrm{anthocyanin}\;(\mathrm{mg}\;C3\mathrm{GE}/L)\;=\;\frac{(A\;\times \;\mathrm{Mw}\;\times \;100)}{ƹ\times 1}$$

A = (Abs_520_nm-Abs_700_nm) pH1 − (Abs_520_nm-Abs_700_nm) pH4.5,
MW = molecular weight (449.2 g/mol for cyanidin-3-glucoside)
DF = dilution factor, 1000 = factor for conversion from g to mg
ƹ = molar extinction coefficient (26,900) in L mol^−1^ cm^−1^
1 = path length in cm.

### 4.3. Preparation of Niosome Formulation

Preparation of Niosome Encapsulate Anthocyanin from Purple Waxy Corn Cob Extract

The extracts with the highest anthocyanin content and antioxidant activity were selected for niosome preparation. The surfactants were used Span 20 (0.035, 0.070%) and cholesterol ratios (0.039, 0.078%), with 10% propylene glycol in phosphate buffer (pH 5.5), and varying concentrations of extract (0.50, 0.75, 1.00% *w*/*v*) were performed in the production method as indicated in Table 5 [38].

### 4.4. The Characterization of Niosome Properties

#### 4.4.1. Percent Entrapment Efficiency (%EE)

Centrifugation was used for entrapment efficiency (%) calculation. The niosome formulation was centrifuged in a microcentrifuge tube at 5000 rpm for 30 min at 4 °C. The pH-differential method was used to assay anthocyanins in the pellet and supernatant (the pellet was previously dispersed in 50% n-propanol). A microplate reader was used to prepare and determine the optimal dilutions at 520 and 700 nm. The percentage entrapment efficiency was determined using the following formula [39]:

Entrapment efficiency (%) = [(Total − Au)/Total] × 100(2)

where Total is the total anthocyanin added, and Au is the total anthocyanin from unentrapped anthocyanin.

#### 4.4.2. Dynamic Light Scattering (DLS)

The particle size (nm), zeta potential (mV), and polydispersity index (PDI) of the niosomes were measured by dynamic light scattering.

The particle size of the niosomes was measured using a Zetasizer Nano ZS (Malvern Instruments, Malvern, Worcestershire, UK). Before analysis, the samples were diluted to a dilution factor of 1:100 with buffer pH 5.5, and the final measurement was reported as the average of three repeated measurements for each sample as the mean ± SD (nm) [40].

Zetasizer Nano ZS (Malvern Instruments, UK) was utilized to determine the zeta potential for charge calculation of niosomes. The samples were diluted to a dilution factor of 1:100 with distilled water and the final measurement was reported as the average of three repeated measurements for each sample as the mean ± SD (mV).

#### 4.4.3. Transmission Electron Microscopy (TEM)

The morphology of the niosomes was analyzed using a transmission electron microscope (Thermo Scientific, Talos F200X G2, Hillsboro, OR, USA). The niosomes were diluted at a 1:10 ratio with a pH 5.5 buffer. The diluted samples were placed on a copper grid and allowed to dry in air at room temperature. A fixed grid was then placed in the microscope and images were taken at different magnifications [41].

#### 4.4.4. Thermal Stability Testing

Thermal stability of the formulations was evaluated using alternating temperature cycles. The samples were stored at 4 °C and 45 °C, with an alternate temperature every 24 h for a period of seven days. Thermal stress test was conducted two weeks after formulation preparation. % Entrapment efficiency, particle size (nm), zeta potential (mV), and PDI of niosome were determined before and after storage [40].

#### 4.4.5. In Vitro Release Study

Niosomes were evaluated for total anthocyanin release using the Franz cell diffusion method. A dialysis membrane (Spectra/Por^®^, MWCO: 12–14 kD, Spectrum Laboratories, Inc., Rancho Dominguez, CA, USA) was used as the membrane for the release test. Evaluations were performed using a magnetic stirrer at 800 rpm and 37 °C. PBS was used as the support medium at pH 5.5. Samples were taken at 0.5, 1, 2, 4, 6, 8, 12, and 24 h. The total anthocyanin content of the samples was determined using the pH differential method, and the percentage of anthocyanin released was calculated at each sampling point [6].

The in vitro release data were analyzed using the linear regression method (R^2^) to evaluate the kinetics of the anthocyanin release. The analysis applied four models: (1) zero-order, which examines the cumulative release percentage over time; (2) first-order, which assesses the logarithm of the cumulative release percentage against time; (3) Higuchi, which evaluates the cumulative release percentage relative to the square root of time; and (4) Korsmeyer–Peppas, which analyzes the logarithm of the cumulative release percentage versus the logarithm of time [6].

#### 4.4.6. Fourier Transform Infrared Spectroscopy FT-IR Spectroscopy

The niosome were air-dried prior to analysis. An ATR-FTIR spectrometer (Bruker Tensor II, Ettlingen, Baden-Württemberg, Germany) was used to confirm the encapsulation of all niosome and PWCCES. The spectral transmittance was recorded over 64 scans with a resolution of 4 cm^−1^, covering a spectral range of 4000–650 cm^−1^ [6].

### 4.5. UVB-Protection

#### 4.5.1. Assessing Cytotoxicity in HaCaT Cells with the MTT Assay

Following treatment with different formulations, the viability of HaCaT cells was observed. In a 96-well plate, 1.5 × 10^4^ cells/well were seeded in DMEM with 10% FBS, 1% streptomycin (100 mg/mL), and 100 U/mL penicillin. The cells were cultured in an incubator with 5% CO_2_ for 24 h at 37 °C. Test samples were substituted as the medium. The culture media was a negative control. The cells were treated with 5% CO_2_ at 37 °C. After discarding the medium, MTT was applied, and the formazan absorbance at 570 nm was measured with a microplate reader to ensure that the cells were more than 80% viable [42]. The cell viability was determined using the following equation:

% Cell viability = (Absorbance of sample/Absorbance of (-) control) × 100(3)

#### 4.5.2. The UVB Irradiation on HaCaT Cells

96-well plates were employed to plate 1.5 × 10^5^ cells/well of HaCaT cells. PBS (0.5 mL) was added to the cells after the removal of medium. The cells were irradiated with light of that wavelength provided by a Philips UVB 20 W/12 (Amsterdam, The Netherlands) broadband phototherapy lamp for 2 min. The irradiance was measured using a photo radiometer. The microplate was kept on ice throughout irradiation to prevent overheating [42].

#### 4.5.3. Effective UVB Protection for HaCaT Cells

The MTT assay of the formulations to shield HaCaT cells from UVB-induced cytotoxicity was evaluated using the MTT assay. HaCaT cells were treated with PWCC and niosome formulations in culture medium for 24 h at 37 °C with 5% CO_2_, followed by treatment with purple waxy corn cob (0.5–1% *w*/*v*) and niosome formulations (N1, N2, N3, N4 and N5) in culture media. Cell viability was assessed after a 24 h incubation period [43].

### 4.6. Anti-Melanogenesis

#### 4.6.1. Cytotoxicity on the B16F10 Cells by the MTT Assay

The initial plating density of B16F10 cells was 1 × 10^4^ cells per well in 96-well plates supplemented with 10% FBS, 1% streptomycin (100 mg/mL), and penicillin (100 U/mL) in Dulbecco’s modified Eagle’s medium (DMEM). Cells were grown for 24 h at 37 °C and 5% CO_2_. After incubation, the culture medium was removed and replaced with PWCCS (0.5% to 1% *w*/*v*) and niosome formulations (N1, N2, N3, N4 and N5) and cultured in culture medium (10% FBS) to a final concentration of 100 µL/well. PWCCS solution and niosomes served as test samples, whereas culture media alone served as the negative control. The medium was discarded after 48 h of incubation at 37 °C and 5% CO_2_. The MTT assay was used to determine the viability of the cells to over 80%, and the formazan absorbance was detected using a microplate reader at 570 nm [43].

The cell viability was determined using Equation (3) as well as Section 4.5.1.

#### 4.6.2. Melanin Contents

B16F10 cells were seeded in 6-well plates at an initial concentration of 1 × 10^5^ cells/well and incubated for 24 h at 37 °C with 5% CO_2_. PWCCS (0.5% to 1% *w*/*v*) and niosome formulations (N1, N2, N3, N4 and N5) were diluted with culture medium, final volume per well of 1000 µL, with the stimulating of α-MSH (200 nM). The cells were incubated for 48 h under the same conditions. The cells were then washed twice with PBS. The pellets were dissolved for one hour at 80 °C in 1 N NaOH with 10% DMSO. Protein content in the supernatant was quantified using the bicinchoninic acid (BCA) assay method, and melanin content was quantified by measuring the absorbance at 475 nm [43].

#### 4.6.3. Anti-Tyrosinase

B16F10 cells were seeded at a density of 1 × 10^5^ cells/well in 6-well plates and incubated for 24 h. The cells were treated with the samples, which were subsequently incubated at 37 °C and 5% CO_2_ for 48 h. The cells were washed twice in PBS and lysed in 200 µL of 0.1 M PBS (pH 6.8) with 0.1% Triton X-100. The protein content in the lysates was determined using a BCA assay. An aliquot of 100 µg/mL protein was combined with 100 µL of 0.1% l-DOPA in PBS (pH 6.8) and incubated at 37 °C for 20 min. Tyrosinase inhibitory activity was determined by measuring the absorbance at 475 nm using a microplate reader [43].

### 4.7. Statistical Data Analysis

The data were processed using SPSS version KKU, and the mean ± standard deviation (SD) of three replicates for all sets of results were reported (n = 3). One-way analysis of variance (ANOVA) followed by Duncan’s post hoc test was used to determine group differences. A paired-sample *t*-test was used to compare the stability tests. Statistical significance was defined as a *p*-value of less than 0.05.

## 5. Conclusions

This study demonstrated that niosomal formulations incorporating niosome formulation N5 (1% PWCC extract with 0.079% Span 20 and 0.078% cholesterol) exhibited favorable physicochemical stability, sustained release behavior, and promising UVB protection, anti-tyrosinase and anti-melanogenic activities. These findings suggest that such formulations could serve as effective and biocompatible delivery systems for natural cosmeceutical applications targeting hyperpigmentation and UV protection. However, as this work was limited to in vitro evaluations, further in vivo and clinical studies are necessary to confirm the efficacy, safety, and skin penetration behavior of the developed formulations under physiological conditions. Future research should also focus on optimizing large-scale production, enhancing formulation stability, and investigating molecular interactions between niosomes and skin cells to support their potential for commercialization in advanced cosmeceutical products.

Nevertheless, the current study investigated only in vitro systems, and in vivo verification is required to establish the bioavailability, dermal permeation, and long-term stability of these formulations. Subsequent studies should aim at optimizing scale-up production, formulating functionality in conditions of actual use, and investigating synergistic activity in association with other bioactive to best exploit therapeutic and cosmetic potential.

## Figures and Tables

**Table 1:** The Characterization of Anthocyanin PWCCs.

Assay	Yield	TAC	Antioxidant Activities		
Unit	(%)	mg C3GE/g DW	DPPH (IC_50_ μg/mL)	ABTS (IC_50_ μg/mL)	FRAP (mg Fe(II)/g)
PWCC Extract	6.34	3.02 ± 0.81	72.32 ± 9.71	17.78 ± 0.57	4.21 ± 0.10
Vit C/Trolox			17.11 ± 0.18	1.01 ± 0.34	

**Table 2:** The Entrapment Efficiency, Particle Size, Polydispersity (PDI), and Zeta Potential.

Formulation	%EE	Size (nm)	PDI	Zeta (mV)
N1	- ^a^	285.70 ± 17.35 ^d^	0.16 ± 0.04 ^a^	−29.30 ± 4.63 ^a^
N2	84.88 ± 2.11 ^c^	221.80 ± 3.43 ^b^	0.16 ± 0.04 ^a^	−30.70 ± 0.91 ^a^
N3	- ^a^	185.60 ± 20.96 ^a^	0.42 ± 0.12 ^b^	−30.70 ± 0.73 ^a^
N4	83.68 ± 3.18 ^b^	296.50 ± 2.34 ^d^	0.16 ± 0.05 ^a^	−32.10 ± 0.11 ^a^
N5	85.74 ± 1.19 ^c^	248.10 ± 9.72 ^c^	0.14 ± 0.06 ^a^	−30.90 ± 2.89 ^a^

Data are reported as the mean entrapment efficiency values (%EE), particle size, polydispersity index (PDI), and zeta potential of three replicates. Statistically significant differences are denoted by the same column letters (a–d) at *p* < 0.05 using one-way ANOVA.

**Table 3:** Entrapment efficiency, particle size (nm), PDI, and zeta potential (mV) of niosome stability study.

	(%EE)		Size (nm)		PDI		Zeta (mV)	
	Before	After	Before	After	Before	After	Before	After
N3	-	-	164.60 ± 32.96 *	257.80 ± 4.86 *	0.28 ± 0.01	0.24 ± 0.03	−30.43 ± 0.42 *	−44.40 ± 0.36 *
N5	82.65 ± 3.65	80.71 ± 188	265.23 ± 9.03 *	344.03 ± 9.65 *	0.33 ± 0.01 *	0.19 ± 0.02 *	−30.10 ± 0.26 *	−40.30 ± 0.17 *

The data are presented as mean ± SD from three replicates. Statistical significance was determined using a paired *t*-test (* *p* < 0.05) compared between before and after storage under heating–cooling conditions. N3 represents the niosome blank, and N5 represents the niosome encapsulating the PWCC extract (1% PWCC extract).

**Table 4:** The kinetic parameters for anthocyanin release obtained from each model of N5 and 1% PWCC extract solution (PWCCES).

Sample	Zero Order		First Order		Higuchi		Korsmeyer–Peppas		
	R^2^	K_0_	R^2^	K_1_	R^2^	K_H_	R^2^	n	K_p_
N5	0.9742	0.1866	0.9385	0.0605	0.9901	3.1461	0.9864	0.4838	3.2636
PWCCES	0.919	1.149	0.8177	0.0569	0.9662	4.5362	0.9556	0.4800	5.1713

**Table 5:** The Preparation of Niosome Formulations.

Formulation	PWCC Extract (%)	Span 20 (%)	Cholesterol (%)	Propylene Glycol (%)	PBS PH 5.5 (%)	Total (%)
N1	0.000	0.035	0.039	10.000	89.926	100.000
N2	0.500	0.035	0.039	10.000	89.426	100.000
N3	0.000	0.070	0.078	10.000	89.852	100.000
N4	0.750	0.070	0.078	10.000	89.102	100.000
N5	1.000	0.070	0.078	10.000	88.852	100.000

N1 and N3 are blank niosome formulations without PWCC extract (control).

## Data Availability

Data are contained within the article and Supplementary Materials.

## Supplementary Materials

The following are available online at https://www.mdpi.com/article/10.3390/ijms262110586/s1.

## Abbreviations

PWCC: Purple Waxy Corn Cob; C3GE: Cyanidin-3-glucoside; TAC: Total Anthocyanin Content; HPLC: High-Performance Liquid Chromatography; DPPH:2,2-Diphenyl-1-picrylhydrazyl; ABTS:2,2′-azino-bis (3-ethylbenzothiazoline-6-sulfonic acid; FRAP: Ferric Reducing Antioxidant Power; IC_50_: Half-maximal Inhibitory Concentration; EE: Entrapment Efficiency; PBS: Phosphate-Buffered Saline; TEM: Transmission electron microscopy; FTIR: Fourier Transform Infrared Spectroscopy; UVB: Ultraviolet B radiation; HaCaT Cell: Human Immortalized Keratinocytes; B16F10 Cell: B16 murine melanoma cell.
