# Supplementary material for: Development of Niosome-Entrapped Purple Waxy Corn Cobs (Zea mays L.) Extracts to Enhance UVB-Protection and Anti-Melanogenesis Activities
Source: Int J Mol Sci. 2025 Oct 30;26(21):10586. doi: 10.3390/ijms262110586 (PMC12607531; doi:10.3390/ijms262110586)
Supplement: Supplementary file 1 [file ijms-26-10586-s001.zip › ijms-3859015-supplementary.pdf]

Supplementary Materials

1. Supplementary Figures and Tables

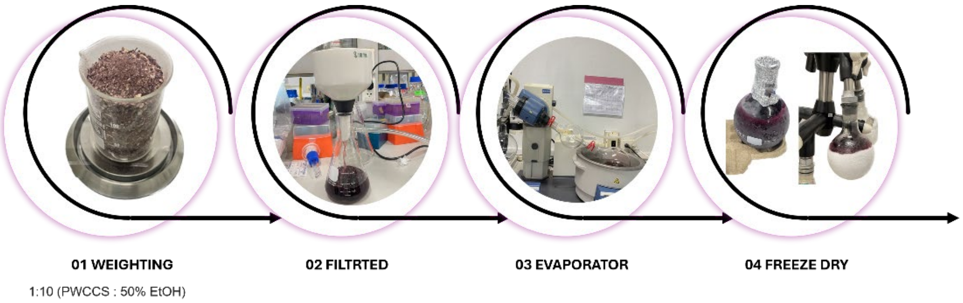

Figure S1 The extraction of anthocyanins from purple waxy corn cob with 50% ethanol by the maceration method [1].

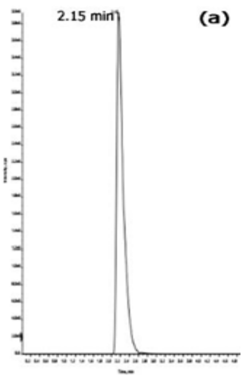

Figure S2 : LC-MS/MS chromatogram of Cyanidine-3-Glucoside (C3G) from purple waxy corn con extract 1 ug/mL determined at m/z at 449/287 [2]. The retention time of C3G is 2.15 min.

Determination of C3G

The validated HPLC-MS/MS procedure was performed for anthocyanin determination using a triple quadrupole machine (API 3200 MS/MS System, ABSciex) equipped with a binary HPLC pump (Hewlett-Packard 1100, Series HPLC Value System) with Analyst software, and a column (Poroshell 120 SB-C18 [4.6 × 75 mm, 2.7 µm]) with a drop-in guard cartridge (Agilent Technologies, USA). Two mobile phases, 5% formic acid (phase A) and methanol (phase B), were subjected to a series of gradient elution, as follows: 0 to 10 minutes, 20% to 30% of phase B, 10 to 13 minutes, 30% to 100% of phase

B, and 13 to 15 minutes, isocratic at 100% of phase B, and then a re-equilibration period of 3 minutes with 20% of phase B between individual runs. Operating conditions were as follows: a flow rate of 0.3 mL/min, a controlled column temperature of 30°C, the sample temperature at ambient, and an injection volume of 0.01 mL. MS parameters were as follows: ionization mode with ESI positive mode, a scan range of 400 to 500 amu, and a scan rate of 1 scan/sec. The source parameters were turbo ion spray voltage of 5 500 V, source temperature (TEM) 550°C, TEM of nebulizer gas (gas 1) 50°C, TEM of heater gas (gas 2) 60°C, curtain gas pressure 25 psi, and collision gas pressure 5 psi. For MS2 analysis, the collision energy and the collision cell exit potential were used at 5 to 130 V and 0 to 58 V, respectively, with a scan range of 400 to 500 amu [3].

Reference

1. Chuntakaruk, H.; Kongtawelert, P.; Pothacharoen, P. Chondroprotective Effects of Purple Corn Anthocyanins on Advanced Glycation End Products Induction through Suppression of NF-κB and MAPK Signaling. *Sci. Rep.* **2021**, *11*, 1895, doi:10.1038/s41598-021-81384-4.
2. Rimdusit, T.; Thapphasaraphong, S.; Puthongking, P.; Priprem, A. Effects of Anthocyanins and Melatonin From Purple Waxy Corn By-Products on Collagen Production by Cultured Human Fibroblasts. *Nat. Prod. Commun.* **2019**, *2019*, 1–6, doi:10.1177/1934578X19863510.
3. Rimdusit, T.; Thapphasaraphong, S.; Puthongking, P.; Priprem, A. Effects of Anthocyanins and Melatonin From Purple Waxy Corn By-Products on Collagen Production by Cultured Human Fibroblasts. *Nat. Prod. Commun.* **2019**, *14*, 1934578X19863510, doi:10.1177/1934578X19863510.
4. Damrongrungruang, T.; Paphangkorakit, J.; Limsitthichaikoon, S.; Khampaenjiraroch, B.; Davies, M.J.; Sungthong, B.; Priprem, A. Anthocyanin Complex Niosome Gel Accelerates Oral Wound Healing: In Vitro and Clinical Studies. *Nanomedicine Nanotechnol. Biol. Med.* **2021**, *37*, 102423, doi:10.1016/j.nano.2021.102423.
5. Nasirian, H.; TarvijEslami, S.; Ghourchian, H.; Ebrahimi, M.; Piri-Gharaghie, T.; Ghajari, G. Niosomes Containing Enciprazine Hydrochloride Have Been Shown to Efficiently Inhibit the Proliferation and Induce Apoptosis in Colorectal Cancer Cells. *Adv. Cancer Biol. - Metastasis* **2024**, *12*, 100128, doi:10.1016/j.adcanc.2024.100128.
6. Muzzalupo, R.; Tavano, L. Niosomal Drug Delivery for Transdermal Targeting: Recent Advances. *Res. Rep. Transdermal Drug Deliv.* **2015**, 23, doi:10.2147/RRTD.S64773.

The method validation using linearity, precision, accuracy, and recovery was performed (Table S1). The extracted samples were subjected to HPLC-MS/MS and the C3G contents were compared with standard curves.

Table S1 Validation method of anthocyanins (C3G) by HPLC-MS/MS [3].

Parameters	C3G
Linearity (n=5)	
Intra-day	
- linear equation	
- coefficient of	Y=1680.2X +12323
determination (R^2)	0.998
Inter-day	
- linear equation	
- coefficient of	Y=1718.1X +31374
determination(R^2)	0.998
Range (ng/mL)	15-800
LOD (ng/mL) (n=7)	0.5
LOQ 9ng/mL) (n=7)	2.0
Precision (%RSD) [a] (n= 7)	
-Intra-day	0.67-4.02 (<10)
-Inter-day	
Accuracy (%RE) [b] (n=5)	
-Intra-day	0.15-15.57 (<20)
-Inter-day	0.90-11.67 (<20)

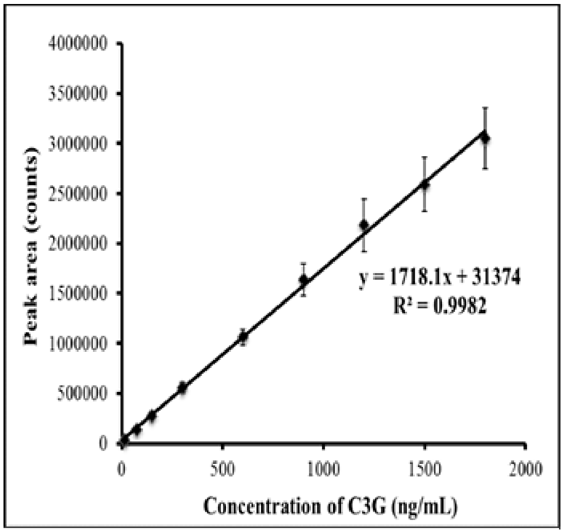

Figure S3 Standard curve of cyanidin-3-glucoside equivalents (C3G)

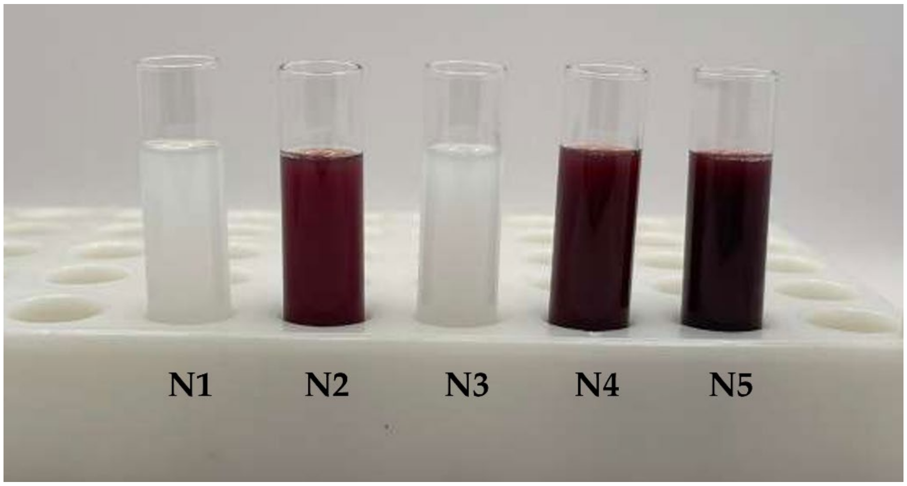

Figure S4 The five niosome formulations (N1-N5) with different concentrations of PWCC extract and ratios of Span20 and Cholesterol [4].

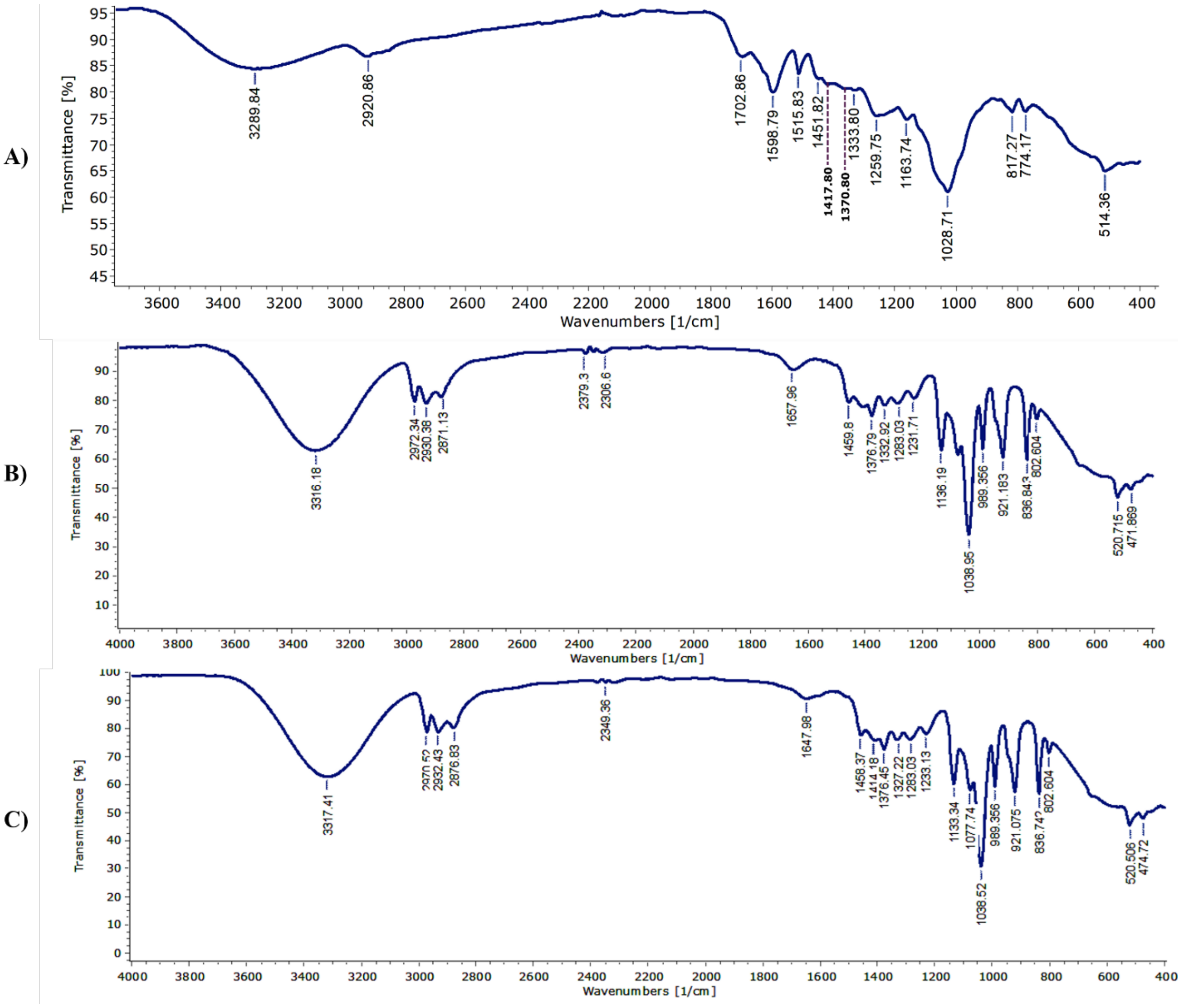

Figure S5 The FTIR spectra of niosome prepared by the Sonication method. (A) PWCC extract, B) Blank niosome (N3), and (C) niosome encapsulating PWCC extract (N5).

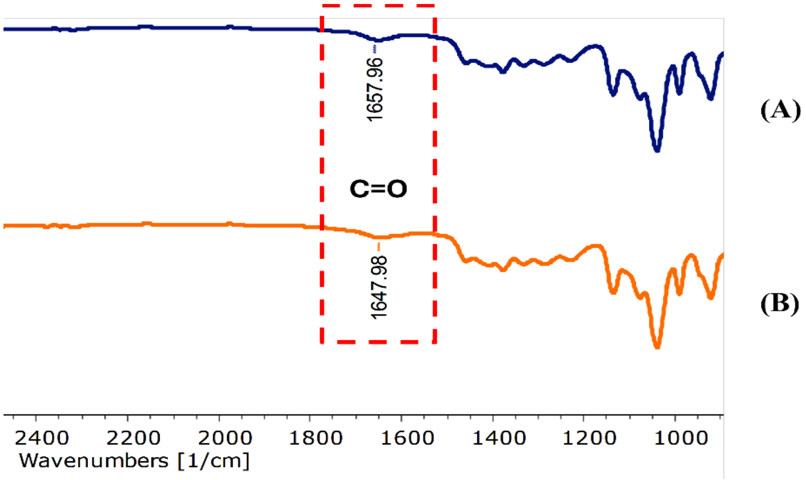

Figure S6 The spectral interactions between the carbonyl functional groups. (A) niosome blank (N3) and (B) niosome encapsulating PWCC extract (N5).

Wavenumber (cm^{-1})			Functional groups
Niosome Blank	Niosome PWCC extract	PWCC extract	
3316.18	3317.41	3289.84	O-H stretching
2972.34<br>2930.38<br>2871.13<br>2379.30<br>2306.60	2970.52<br>2932.43<br>2876.83<br>2349.36	2920.86	C-H stretching
**1657.96**	**1647.98**	**1702.86**	C=O stretching
-	-	1598.79<br>1515.83	C=C stretching
1459.80	1458.37<br>1414.18	1451.82<br>1417.80	C-C stretching
1376.79<br>1332.92	1376.45<br>1327.22	1370.80<br>1333.80	CH2 bending
1283.03<br>1231.71<br>1136.19<br>1038.95	1283.03<br>1233.13<br>1133.34<br>1077.74<br>1038.52	1259.75<br>1163.74<br>1259.75<br>1163.74	C-O stretching
989.356<br>921.183<br>836.843<br>802.604<br>520.715<br>471.869	989.356<br>921.075<br>836.742<br>802.604<br>520.506<br>474.720	817.27<br>774.17<br>514.36	C-H bending

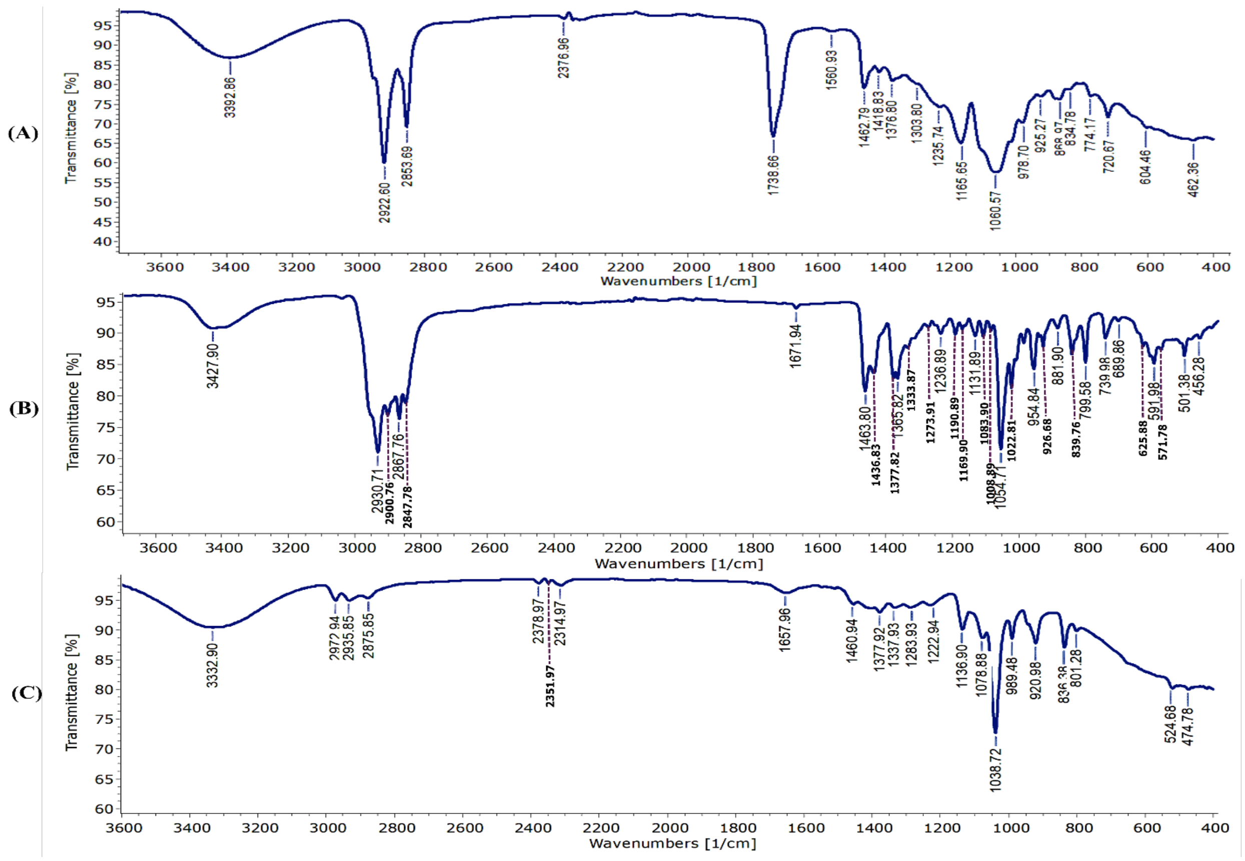

Figure S7 The FTIR spectra of Span20, Cholesterol, and Propylene Glycol [5].

Wavenumber (cm^{-1})			Functional groups
Span20	Cholesterol	Propylene Glycol	
3392.86	3427.90	3332.90	O-H stretching
2922.60<br>2853.69<br>2376.96	2930.71<br>2900.76<br>2847.78	2972.94<br>2935.85<br>2875.85<br>2378.97<br>2351.97<br>2314.97	C-H stretching
1738.66	1671.94	1657.96	C=O stretching
1560.93	-	-	C=C stretching
1462.79<br>1418.83	1463.80<br>1436.83	1460.94	C-C stretching
1376.80<br>1303.80	1377.82<br>1365.82<br>1333.87	1377.92<br>1337.93	CH2 bending

Wavenumber (cm^{-1})			Functional groups
Span20	Cholesterol	Propylene Glycol	
1235.74	1273.91	1283.93	C-O stretching
1165.65	1236.89	1222.94	
1060.57	1190.89	1136.90	
	1169.90	1078.88	
	1131.89	1038.72	
	1008.89		
	1054.71		
	1022.81		
978.70	954.84	989.48	C-H bending
925.27	926.68	920.98	
868.97	881.90	836.38	
834.78	839.76	801.28	
774.17	799.58	524.68	
720.67	739.98	474.78	
604.46	689.86		
462.36	625.88		
	591.98		
	571.78		
	501.38		
	456.28		

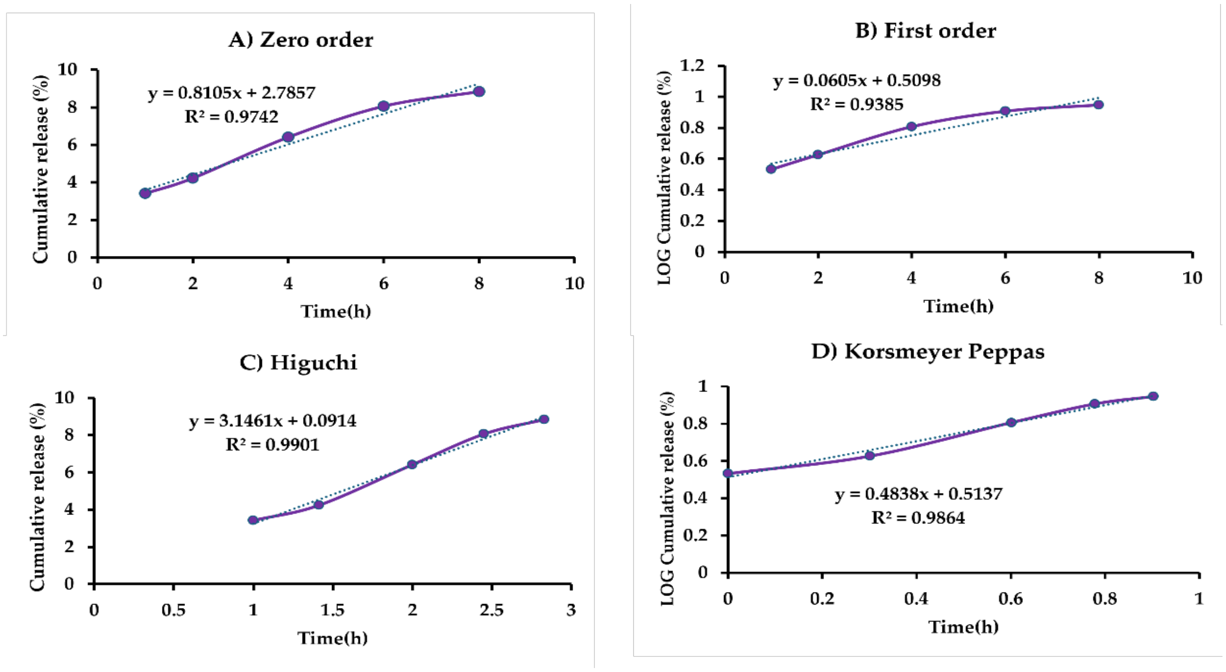

Figure S8 The kinetic model prediction of release for N5 (1% PWCC extract with 0.070% Span 20 and 0.078% cholesterol) [6].

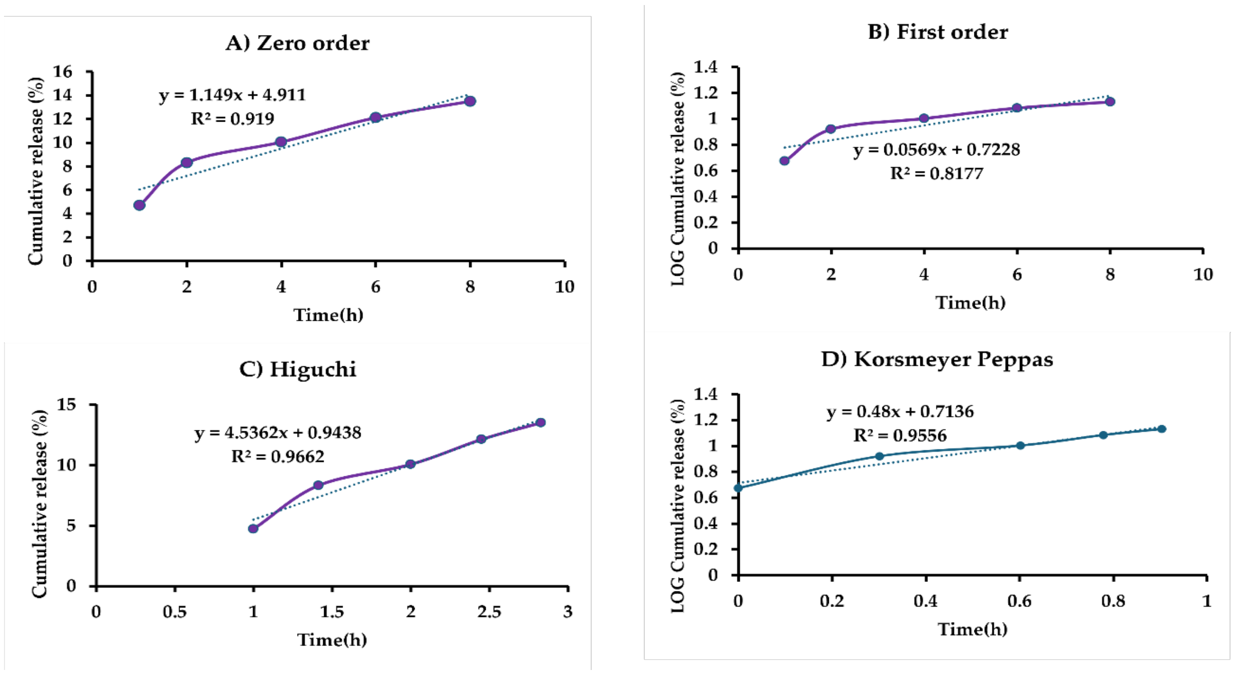

Figure S9 The kinetic model prediction of release for PWCCES (1% PWCC extract with 0.070% Span 20 and 0.078% cholesterol) [6].

Table S2 Cumulative release of Anthocyanins from PWCCS for N5 and 1% PWCC extract solution (PWCCES).

Cumulative release of anthocyanins				
Time (h)	N5	PWCCES	SD of N5	SD of PWCCES
0.5	0.782	2.472	0.051	0.158
1	3.41	4.711	0.019	0.204
2	4.231	8.315	0.088	0.184
4	6.411	10.055	0.102	0.168
6	8.061	12.114	0.186	0.051
8	8.837	13.49	0.088	0.27
12	9.654	15.27	0.135	0.019
24	10.668	17.322	0.07	0.171

The calculated value of Korsmeyer–Peppas model.
Equation:

$$\frac{M_t}{M_\infty} = K_P\, t^n$$

or in logarithmic form:

$$\mathbf{log}\,(\frac{\boldsymbol{M_t}}{\boldsymbol{M_\infty}}) = \mathbf{log}\ \boldsymbol{K_P} + \boldsymbol{n}\mathbf{log}\ \boldsymbol{t}$$

Where:

- M_t/M_∞= fraction of drug released at time t
- K_P= Release rate constant
- n= release exponent (indicates the mechanism of release)

How to calculate:

- Plot **log(M_t/M_∞)** vs. **log(t)**.
- The **slope** gives **n** (release exponent).
- **The intercept gives log(K_p), so:**

$$K_P = 10^{\text{intercept}}$$

Interpretation of n (for a cylinder/sphere):

- n ≅ 0.5: Fickian diffusion (pure diffusion).
- 0.5 < n < 1.0 : Anomalous transport (diffusion and relaxation/erosion occurring concurrently).
- n ≅ 1.0: Case II transport (release governed primarily by polymer chain relaxation/erosion).

The Korsmeyer–Peppas constant (Kp), R^2 and n were obtained from the plot between logarithm of the cumulative release percentage versus the logarithm of time (Figure S8D and Figure S9D). The linear equation provides R^2 , n is from slope of the equation and Kp is equal to $10^{\text{intercept}}$.

Calculation:

1) From linear equations of N5
   Y= 0.4838x + 0.5137
   So, Kp = $10^{0.5137}$ = 3.2636
2) From linear equations of PWCCES
   Y = 0.48x + 0.7136
   So, Kp = $10^{0.5137}$ = 5.1713

The Korsmeyer–Peppas release rate constant (K_p) represents the overall rate at which the active compound diffuses or is released from the niosomal matrix. Therefore, Kp of N5 and PWCCES were 3.2636 and 5.1713 respectively. PWCCES has a higher K_p value indicates a faster release rate of the entrapped compound, whereas N5 has a lower K_p,

suggesting a slower and more sustained release profile. Therefore, K_p can be used to compare the release performance among different formulations, reflecting how formulation parameters influence drug diffusion through the vesicular system.

Furthermore, the release exponent(n) obtained from Korsmeyer-Peppas model describes the mechanism of drug release from delivery system. In this study, n value of N5 (0.4838) was close to 0.5, indicating the release of anthocyanins followed Fickian diffusion mechanism.

Table S3 Summary of Drug Release Kinetic Models

Model	Equation	Plot	parameters	Interpretation
Zero-order	Qt=K0t	Qt vs. t	Slope = K_0	Constant release rate over time
First-order	log(100−Qt)=log100−(K_1t/2.303)	log(100−Qt) vs. t	Slope = $-K_1/2.303$ $\rightarrow K_1 = -2.303 \times$ slope	Release rate depends on remaining drug
Higuchi	Qt=$K_H\sqrt{t}$	Qt vs. $\sqrt{t}$	Slope = K_H	Diffusion-controlled release
Korsmeyer–Peppas	log(Mt/M∞) =logKp + nlogt	log(Mt/M∞) vs. log t	Slope = n; Intercept = log K_P	Determines mechanism (diffusion, anomalous, etc.)

Q = cumulative % release at time t

M_t/M_∞= fraction of drug released at time t
